# A Versatile Processing Workflow to Enable Pathogen Detection in Clinical Samples from Organs Using VIDISCA

**DOI:** 10.3390/diagnostics11050791

**Published:** 2021-04-27

**Authors:** Alba Folgueiras-González, Robin van den Braak, Martin Deijs, Lia van der Hoek, Ad de Groof

**Affiliations:** 1 Department Discovery & Technology, MSD Animal Health, Wim de Körverstraat 35, P.O. Box 31, 5830 AA Boxmeer, The Netherlands; alba.folgueiras.gonzalez@merck.com (A.F.-G.); robin.braak.van.den@merck.com (R.v.d.B.); 2 Laboratory of Experimental Virology, Department of Medical Microbiology, Amsterdam UMC Location AMC, University of Amsterdam, Meibergdreef 9, 1105 AZ Amsterdam, The Netherlands; m.deijs@amsterdamumc.nl (M.D.); c.m.vanderhoek@amsterdamumc.nl (L.v.d.H.)

**Keywords:** virus discovery, metagenomics, VIDISCA-NGS, atypical porcine pestivirus, enrichment

## Abstract

In recent years, refined molecular methods coupled with powerful high throughput sequencing technologies have increased the potential of virus discovery in clinical samples. However, host genetic material remains a complicating factor that interferes with discovery of novel viruses in solid tissue samples as the relative abundance of the virus material is low. Physical enrichment processing methods, although usually complicated, labor-intensive, and costly, have proven to be successful for improving sensitivity of virus detection in complex samples. In order to further increase detectability, we studied the application of fast and simple high-throughput virus enrichment methods on tissue homogenates. Probe sonication in high EDTA concentrations, organic extraction with Vertrel™ XF, or a combination of both, were applied prior to chromatography-like enrichment using Capto™ Core 700 resin, after which effects on virus detection sensitivity by the VIDISCA method were determined. Sonication in the presence of high concentrations of EDTA showed the best performance with an increased proportion of viral reads, up to 9.4 times, yet minimal effect on the host background signal. When this sonication procedure in high EDTA concentrations was followed by organic extraction with Vertrel™ XF and two rounds of core bead chromatography enrichment, an increase up to 10.5 times in the proportion of viral reads in the processed samples was achieved, with reduction of host background sequencing. We present a simple and semi-high-throughput method that can be used to enrich homogenized tissue samples for viral reads.

## 1. Introduction

Despite the advances in diagnostics in the last decades, there are still a variety of veterinary and human diseases of unknown etiology, many of which are suspected to have a viral origin [1,2,3]. Characterization of viral involvement is complicated by the inability of many viruses to replicate in vitro, by lack of sequence information to develop precise amplification PCRs, and by difficulties raising specific antibodies that enable virus detection. Improved molecular methods such as universal PCR primers [4], random priming PCR [5], or representational difference analysis (RDA) [6], together with the introduction of high throughput sequencing, have increased the prospects for discovery and detection of known and novel viruses without prior knowledge on their genome sequence, even when present in low viral loads. One of these unbiased virus discovery methods is VIDISCA, virus discovery cDNA-AFLP (amplified fragment length polymorphism) [7]. VIDISCA relies on viral dsDNA genome fragmentation using MseI, a frequent-cutting restriction enzyme of which recognition sites are present in high abundance in viral genomes, and subsequent adaptor ligation onto the sticky ends followed by PCR amplification. The library of PCR products can be sequenced via the Ion Torrent PGM platform. A characteristic of VIDISCA is the low number of sequences needed to detect (novel) viruses: 5000–10,000 sequence reads per sample [8]. The method has been successfully used for detecting a large number of novel human and animal viruses in cell culture supernatants [9], sera [10], feces [11], respiratory clinical samples [7], and cerebrospinal fluid [8].

The low relative abundance of genetic material of viruses in a diagnostic sample compared to that of the host organism is still a crucial aspect when performing viral metagenomics. This becomes an even bigger bottleneck when using tissue homogenates, which contain a plentiful and complex background of contaminating host genetic material, mainly ribosomal RNA (rRNA) in ribosomes that cannot be separated from viruses by size selection or eliminated with nuclease treatments [12,13]. Sophisticated methods have been developed in recent years, e.g., cDNA microarrays [14], RDA [6], or ultradeep sequencing [15], but their cost and required expertise constrain their use to only a few research facilities. Physical enrichment processing methods can also improve the sensitivity of virus detection when used prior to metagenomic sequencing. However, these methods are sample specific, sequencing-platform specific, or rely on laborious ultracentrifugation or density-gradient separation that cannot be used in semi-high-throughput settings [16].

Novel virus identification is not always feasible in sera or body fluids because the pathogen might not be present in these samples. In some cases, viruses may only be detectable in the tissues where they replicate and cause disease [17,18]. Given the fact that a large number of novel viruses cannot be cultured in vitro from organ homogenates because the appropriate cell substrate is not known, the physical processing of viruses directly from tissues becomes a critical step prior to sequencing for the discovery of such novel pathogens. A pre-processing protocol aimed at a general application should therefore ideally increase the proportion of viral reads while reducing the host background and should, at the same time, be simple, cost-effective, fast, sensitive, and robust.

Ribosomal RNA accounts for around 80% of total cellular RNA. Stably located within the ribosome structure, accessing this RNA constitutes a particularly difficult hurdle to overcome when performing metagenomics for RNA virus discovery [7,13]. Thus, when developing a sample processing method for improved virus discovery, rRNA would be the first target element to deplete. However, ribosomes and viruses have a similar structure and size so are both likely retained in the same fraction when purifying samples using physical processing methods. Meanwhile, other background material present in the host cells, such as mitochondrial RNA, non-coding RNA, or host messenger RNA, also act as competing nucleic acid sequences, and their depletion will also liberate sequencing space for virus discovery using NGS.

Here, we present a proof-of-concept study for the development of a novel semi-high-throughput processing approach for the enrichment and detection of viral genome sequences in tissue homogenates. Organ samples from piglets infected with atypical porcine pestivirus (APPV) were processed in four distinct experimental workflows adapted from a research study from Shmulevitz and co-workers [19]. We tested the efficacy of tissue disruption using sonication in combination with high EDTA concentrations, previously proven useful for the denaturation of ribosomes [20], and organic extraction with Vertrel™ XF, a Freon substitute optimized for virus extraction [21]. Subsequently, Capto™ Core 700, a commercially available chromatography resin based on beads bearing a ligand-activated core that entraps only molecules smaller than 700 kDa, was used for further enrichment of viral particles using an in-slurry approach. To date, Capto™ Core 700 chromatography has proven to be effective in purifying, among others, influenza A and B viruses [22], reovirus [19], respiratory syncytial virus [23], and virus-like particles [24,25,26].

## 2. Materials and Methods

### 2.1. Clinical Samples

Tonsils were taken from a previous published experimental study where postmortem examination was performed on newborn piglets with clinical symptoms of congenital tremor type A-II that were positive for atypical porcine pestivirus (APPV) after infecting pregnant gilts at day 32 of gestation [10]. Organs were sampled and frozen at −70 °C until analysis. Homogenization was performed in PBS (1:10 *w*/*v*) using Gentle Macs M tubes with the Gentle Macs Dissociator (Miltenyi Biotec, Bergisch Gladbach, Germany) following the manufacturer’s manual.

Each replicate was performed with a batch of tonsil homogenate originating from three piglets from the same litter affected by congenital tremor type A-II that were positive for atypical porcine pestivirus [10]. Different litters were used for each of the replicates. Homogenates within each replicate were pooled and aliquoted for further processing.

### 2.2. Sample Processing Workflow and Experimental Set Up

Four experimental processing workflows were performed on 5 mL aliquots of the pooled tonsil homogenates as specified in Table 1. EDTA and Vertrel™ XF (DuPont Fluorochemicals, Wilmington, DE, USA) were added in each of the cases prior to sonication.

Probe sonication was performed using a Bandelin Sonopuls HD 2200 ultrasonic homogenizer (Bandelin, Berlin, Germany). Homogenates were sonicated on ice, at 70% amplitude for eight pulses of 10 s each, with a 30 s pause in between pulses. Homogenates were then centrifuge at 3200× *g* for 30 min at 4 °C. Supernatant was collected and transferred to clean tubes. In the samples treated with Vertrel™ XF, a top aqueous phase and a bottom organic phase were formed and only the aqueous phase was collected. Two hundred microliters of the supernatant from each processing workflow were stored for further molecular analysis and the rest was used for subsequent Capto™ Core 700 chromatography.

To prepare the Capto™ Core 700 slurry (GE Healthcare Life Sciences, Uppsala, Sweden), the resin was centrifuged at 800× *g* for 5 min. Supernatant was removed and the beads were washed three times with an equal volume of Virus Dilution Buffer (NaCl 150 mM, MgCl_2_ 15 mM, Tris 10 mM, pH 7.4, 0.2 µm filtered-sterilized). The Capto™ Core 700 slurry was stored as 50% ready-to-use dilution in Virus Dilution Buffer at 4 °C.

The ready-to-use Capto™ Core 700 slurry was added to the supernatants collected from the different experimental processing workflows making up to 20% of the final sample volume. The samples were mixed end-over-end using a vertical tube rotator for 45 min at 4 °C. Samples were subsequently centrifuged at 800× *g* for 5 min at 4 °C and the supernatant transferred to clean tubes. This exact procedure was repeated for another two Capto™ Core 700 rounds. Two hundred microliters of the supernatant from each processing workflow were stored for further molecular analysis after each of the chromatography rounds.

We acknowledge that a slight dilution took place with the processing (1.6-fold dilution in workflows 2 and 4) and during each chromatography round (1.125-fold dilution). Therefore, correction on volumes was performed in the following qPCR analysis results. VIDISCA is not a quantitative method, thus, results of VIDISCA-NGS have not been corrected for volume changes.

### 2.3. Atypical Porcine Pestivirus (APPV) and Ribosomal RNA (rRNA) Detection in Clinical Samples by RT-qPCR

Nucleic acids were extracted by the Boom extraction method [27] and subsequent reverse transcription was performed using non-ribosomal random hexamers [28]. A universal quantitative reverse transcription PCR (qRT-PCR) was used to quantitatively detect APPV on the samples as previously described [29].

A RT-qPCR was designed for the relative quantification of rRNA. The qPCR reactions were performed on a final volume of 20 μL containing 10 μL 2× QuantiTect Probe RT-PCR Master Mix (Qiagen, Hilden, Germany), 0.2 μL QuantiTect RT Mix (Qiagen, Hilden, Germany), 6.05 μL Water, 0.5 μL rRNA forward 10 μM primer (rRNA_F: AAACAAAGCATCGCGAAGGC), 0.5 μL rRNA reverse 10 μM primer (rRNA_R: CGCTTCATTGAATTTCTTCACTT), 0.25 μL 10 μM probe (6FAM-GGGTGTTGACGCGATGTGATTTCT-TAMRA), and 2.5 μL of template RNA. Thermocycling was performed in a Rotor-Gene Q (Qiagen, Hilden, Germany) real-time PCR instrument starting with a reverse transcription step of 30 min at 50 °C and a PCR-initial heat activation step of 15 min at 95 °C, followed by 45 cycles of 15 s at 95 °C (denaturation) and 60 s at 60 °C (annealing and elongation). Results were analyzed with the Q-Rex software v1.0 (Qiagen, Hilden, Germany). Positive and negative controls were included in every run.

### 2.4. VIDISCA-NGS

Samples collected after each processing and enrichment round, as well as the starting material, were input for library preparation by VIDISCA and tested by next generation sequencing, as described by de Vries et al. [7]. Briefly, samples were centrifuged, and the supernatant was treated with TURBO™ DNase (Thermo Fisher Scientific, Waltham, MA, USA). Nucleic acids were extracted using the Boom method, an isolation procedure based on the lysing and nuclease inactivating properties of the chaotropic agent guanidinium thiocyanate (GuSCN) and the concurrent binding of all nucleic acid types (DNA and RNA, both single and double stranded) by silica particles in the presence of this agent [27]. The reverse transcription reactions were performed using non-ribosomal random hexamers [28]. Subsequently, a second DNA strand was synthesized, purified, and digested with MseI (T^TAA; New England Biolabs, Ipswich, MA, USA). Adaptors incorporating a sample identifier sequence were ligated to the digested fragments. To remove the small DNA fragments before PCR amplification, the fragments were purified by size selection with AMPure XP beads (Beckman Coulter, Brea, CA, USA) that select for DNA strands with lengths between 100 and 400 bp. A 28-cycle PCR with adaptor-annealing primers was then performed on the purified fragments as described previously [7]. Quant-it dsDNA HS Qubit kit (Thermo Fisher Scientific Waltham, MA, USA) was used to quantify the DNA concentration, while the Bioanalyzer (High Sensitivity Kit, Agilent Genomics, Santa Clara, CA, USA) was used to determine the average nucleotide lengths of the libraries. Seventy sample libraries were pooled at the equimolar concentration as described previously [7,8,11]. Sequencing was performed with the Ion Torrent PGM™ platform (Thermo Fisher Scientific, Waltham, MA, USA) using the ION 316 Chip (400 bp read length and 2 million sequences per run). Viral read confirmation as APPV or rRNA was established when the original VIDISCA read could be aligned to a reference sequence of the virus or rRNA with a nucleotide identity of at least 80% using CodonCode Aligner (version 6.0.2). The number of reads aligned to a reference sequence in CodonCode Aligner was taken as the number of viral reads per sample.

### 2.5. Enzyme Activity Validation

The effect of Vertrel™ XF organic extraction, EDTA treatment, and Vertrel™ XF organic extraction combined with EDTA treatment on the DNAse activity of the TURBO™ DNase reaction was tested on a Lambda DNA HindIII digested marker (4 µg/reaction) (Thermo Fisher Scientific, Waltham, MA, USA). The effect was analyzed by means of gel electrophoresis before and after nucleic acid extraction using the Boom method [27]. Experiments were performed in triplicate.

Samples after Vertrel™ XF organic extraction, EDTA treatment and Vertrel™ XF organic extraction combined with EDTA treatment containing the RNA virus HCoV-NL63 were evaluated for efficiency of the Boom extraction and subsequent reverse transcription. Samples were treated with the different experimental conditions. Thereafter, Boom extraction was performed, and samples were analyzed by RT-qPCR (63NFW: AAACCTCGTTGG AAGCGTGT; 63NRV: CTGTGGAAAACCTTTGGCATC; 63NProbe: FAM-ATGTTATTCAGTGCTTTGGTCCTCGTGAT) [30]. Experiments were performed in triplicate. Statistical analysis was done using GraphPad Prism v.8.1.1 (GraphPad Software, San Diego, CA, USA).

## 3. Results

### 3.1. Identification of Atypical Porcine Pestivirus (APPV) and Ribosomal RNA in Processed Tissue Material by RT-qPCR

We tested four tissue homogenate processing workflows (Table 1), followed by either no, one, two or three rounds of enrichment using Capto™ Core 700 slurry. After each processing or enrichment step, samples were taken for quantification of APPV and rRNA by RT-qPCR, as well as by VIDISCA-NGS analysis. Table 2 shows the qPCR results for two experimental replicates of each complete workflow.

After processing of the homogenates and, in the respective cases, enrichment with Capto™ Core 700 slurry, APPV could be detected by qPCR in all samples. A slight-to-moderate reduction of APPV genome copy numbers compared to the starting material was visible in all workflows prior to Capto™ Core 700 chromatography. Notably, workflow 4 did lead to a substantial decrease of APPV genome copies/µL, particularly after the first enrichment round (103-times decrease in replicate 2). These results suggest that the combined action of EDTA 25 mM and Vertrel™ XF either affects the integrity of the virus particles, which subsequently leads to their degradation, release of viral RNA, and capture of the viral RNA by the Capto™ Core 700 beads, or removes non-encapsidated viral RNA present in the sample.

Background rRNA was also measured via RT-qPCR in the processed samples and expressed in cycle quantification (Cq) units. Fold reduction was calculated using the Cq values as $\alpha^{∆Cq},$ where *α* is the qPCR efficiency and ∆Cq is the difference in Cq value between two different chromatography rounds. An initial reduction in rRNA Cq value compared to the starting material was noticed in workflows 2, 3, and 4 prior to Capto™ Core 700. In workflows 1 and 3, there was no subsequent decrease of rRNA (increase in Cq values) during the three subsequent Capto™ Core 700 rounds (See Table 2). Of note, homogenates processed through the Vertrel™ XF organic extraction in workflow 3 showed fluctuating values for rRNA, around 1 to 2 Cq above the initial value of the homogenized tissue (mean 2.21-fold rRNA decrease after three bead chromatography rounds). As shown in Table 2, homogenates processed using workflows 2 and 4 showed an overall decrease of rRNA, represented by an increase of up to 10 Cq values after processing and enrichment (replicate 2, workflow 4 after three chromatography rounds), compared to the initial tissue homogenate (mean increase of 7.83 Cq values after 3 chromatography rounds). The use of Vertrel™ XF extraction in combination with 25 mM of EDTA showed a very noticeable reduction on rRNA quantity, with a 48.50-fold decrease (replicate 1) and 1305.15-fold decrease (replicate 2) between the sample after three Capto™ Core 700 chromatography rounds and that of the initial homogenate. It has to be noted however, that the very high loads of rRNA in the materials (Cq value < 15) might have led to disturbances on the qPCR results and thus compromised the accuracy of its quantification [31].

Samples with Vertrel™ XF, EDTA, and Vertrel™ XF combined with EDTA containing the RNA virus HCoV-NL63 were evaluated for efficiency of Boom extraction and subsequent reverse transcription. The results showed no statistically significant difference between the treatment conditions and the control (Supplementary Figure S1; One-way ANOVA; *p*-value = 0.8886). In conclusion, no adverse effect of EDTA and Vertrel™ XF treatments on RT-qPCR activity was observed, indicating that these treatments are not related to lower read numbers in the workflows where they were applied.

### 3.2. Effect of Pre-Processing of Organ Homogenates in the Detection of Viruses in VIDISCA-NGS

Although ribosomal RNA is considered the major sequence competitor for virus discovery in NGS platforms based on viral reads, other genetic material, such as host genomic DNA and mRNA, mitochondrial DNA, tRNA, and other non-coding RNAs also constitute an important source of background in next generation sequencing platforms such as VIDISCA-NGS. In an ideal situation, a reduction of background sequences would lead to an increase in the percentage of viral reads among the total number of reads, thereby improving the sensitivity. Therefore, VIDISCA-NGS was performed on a sample from every workflow after each of the processing rounds. Table 3 shows the number and proportion of rRNA reads (as a sum of 18S and 28S rRNA reads) and APPV sequence reads identified in VIDISCA-NGS.

The total number of rRNA reads did not decrease with pre-processing of the tissue homogenates nor, importantly, with repeated bead chromatography. In fact, and rather surprisingly, the percentage of rRNA reads increased up to values over 90%. The most likely explanation for this observation is that despite removal of some of the ribosomal RNA, there is an even stronger reduction of other sources of background DNA and RNA. Workflow 4 is the exception, as it showed a decrease in the number of rRNA reads, but also a significant reduction in the total number of reads that could be generated from this material.

We were surprised to see that VIDISCA was able to retrieve APPV reads from the initial tissue homogenates in each of the two replicates, despite the enormous amount of background of host genetic material in these unpurified materials. Nevertheless, the number of APPV reads accounted for only a minimal proportion (0.006% and 0.013% for replicates 1 and 2, respectively) of the total number of reads detected in the tissue homogenate. Subsequent processing showed a slight increase in sensitivity, especially with workflow 2 (3.5-times in replicate 1 and 9.4-times in replicate 2, Table 3) after pre-treatment of the tissue homogenates with high concentrations of EDTA prior to any bead chromatography round. The absolute number of reads was also increased with workflow 2, in contrast to the other workflows that did not show a substantial improvement in the absolute number of APPV reads over the course of the pre-treatment.

**Figure 1:**
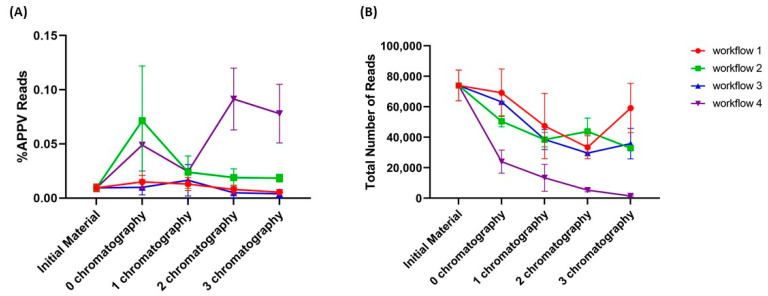
Effect of the novel semi-high-throughput processing workflows on the (**A**) percentage of viral reads and (**B**) total number of reads after each Capto™ Core 700 chromatography round.

A benefit of core bead chromatography was found in workflow 4. After one round of Capto™ Core 700 the proportion of viral reads was 4.2-times higher and 5.6-times higher in replicate 1 and replicate 2, respectively. The percentage of viral reads increased further by the second bead chromatography round to 10.5-times and 9.2-times higher in replicates 1 and 2, respectively (Table 3). Sonication alone (workflow 1) did not result in significant enrichment of viral reads in subsequent chromatography rounds, which indicates that these were largely ineffective. The pre-processing steps with EDTA (workflow 2) and Vertrel™ XF (workflow 3) were aimed at a selective disruption of primarily ribosomal complexes, enabling subsequent core bead chromatography and resulting in a relative increase in the proportion of viral reads compared to the total number of reads (Figure 1A). However, only when the two treatments were combined in workflow 4 were such sensitivity improvements observed, with a substantial decrease on the total number of background reads (Figure 1B).

It is important to note that the absolute number of viral reads decreased after each bead chromatography round (Table 3), in accordance with the data obtained by RT-qPCR. This indicates that only a limited number of Capto™ Core 700 rounds can be applied to achieve enrichment. Furthermore, a reduction in the total number of reads obtained by VIDISCA after processing the samples following workflow 4 and several rounds of core bead chromatography will further decrease the possibility of finding viral sequence reads (see for instance workflow 4, <2000 sequence reads after chromatography round 3). While the resin cleared the sample from <700 kDa non-viral contaminants that were taking up sequencing space, which was indeed supported by an increased percentage of viral reads, the results also showed that the total number of reads became a limiting factor when too many rounds of enrichment are applied. These results indicate that there is likely an optimum to be found in processing, which appears to have been reached after two rounds of Capto™ Core 700 chromatography.

An additional metagenomics experiment was performed in order to check if the selected workflow, using Vertrel™ XF organic extraction and EDTA treatment followed by two Capto™ Core 700 purification rounds, was suitable for the analysis of other types of tissue. Spleen and brain homogenates from the same animals as those from which the tonsils used in the current study originated were analyzed (Supplementary Table S1). qPCR analysis on these homogenates had already revealed that the APPV viral load in these tissues was lower, yet the protocol with two subsequent rounds of Capto™ Core 700 chromatography enrichment allowed detection of APPV via VIDISCA-NGS.

The effect of Vertrel™ XF organic extraction, EDTA treatment, and Vertrel™ XF organic extraction combined with EDTA treatment on the DNAse activity of the TURBO™ DNase reaction was tested on a Lambda DNA HindIII digested marker. We were able to show that in the untreated samples (Supplementary Figure S2, Sample 1) and in the samples extracted using Vertrel™ XF (Supplementary Figure S2, Sample 2) treated with EDTA (Supplementary Figure S2, Sample 3) or pre-processed with a combination of both (Supplementary Figure S2, Sample 4), the TURBO™ DNase was still active and that the DNA was degraded. When no TURBO™ DNase was added to the sample (Supplementary Figure S2, Sample 5), Lambda DNA HindIII fragments were still present. Comparison of Figure S2A,B shows that the exact same results were obtained when samples were checked after the Boom nucleotide extraction. In conclusion, there was no effect of EDTA treatment, Vertrel™ XF organic extraction, or combined pre-processing on DNase enzyme activity.

## 4. Discussion

We investigated if tissue homogenate processing steps such as sonication, with or without high EDTA concentrations and with or without organic extraction with Vertrel™ XF, can improve accessibility of virus particles for subsequent size selection enrichment steps. Such enrichment would theoretically improve NGS-based detection methods for viral nucleotide sequences in homogenates, which currently have a very low success rate. We showed that sonication in the presence of high concentrations of EDTA increases the proportion of viral sequence reads in NGS. In addition to that, we showed that EDTA treatment combined with organic extraction with Vertrel™ XF followed by two rounds of core bead chromatography results in a bigger and substantial increase of up to 10.5 times in the proportion of viral sequence reads that can be identified by NGS in these homogenates. Both processing workflows result in increased sensitivity for finding known, unexpected, and new viruses in solid organ samples.

We hypothesize that the chelating properties of EDTA helped to partially dissociate and denature the ribosome complexes, making the free rRNA more accessible for its potential degradation and/or subsequent removal in the organic fraction [20,32]. EDTA can liberate molecules from cellular complexes and neutralize their charge, which allows for their removal in the extraction with Vertrel™ XF. The EDTA alone may help to make the overall cell and viral material more accessible. When coupled with an organic extraction, the damaged or non-encapsidated virus nucleic acids will likely be lost in the organic fraction, along with the background material present in the host cells. The results suggest that there is likely an optimum to be found in processing, depending on the material used.

The current VIDISCA enrichment methods, i.e., DNase, centrifugation, and non-rRNA-annealing hexamers in reverse transcription [7], coupled with improved library preparation and sequence analysis [33] is sufficient for the retrieval of viral reads when a minimum of 10,000 reads is obtained per sample [7,8,9,10,11], yet, whether this would work for tissue homogenates was unknown. Here we showed that virus concentrations in the range of 10^4^ copies per gram of tissue homogenate can be detected with the standard procedure; however, further enrichment methods as experimentally addressed in the current study have the potential to substantially improve the efficiency.

The viral sample enrichment steps that we applied to homogenized tissue samples are not part of published workflows for discovery of novel viruses using sequence-independent metagenomics. In the veterinary virology field, Delwart et al. introduced a rapid and simple protocol for the identification of known and new viruses, more than a decade ago, based on homogenization of animal tissues, filtration, centrifugation at 22,000× *g* for 2 h, and DNase treatment [34,35]. Since then, their extensive research has demonstrated the possibility of studying the virome in a diverse array of tissues and animal species, e.g., python liver [36], sea lion mesenteric lymph node [37], porcine heart and lung [38], and giant panda heart, liver, spleen, lung, and kidney [39], with minimal modification of the initial physical enrichment methodology. The library preparation (Nextera™ XT library preparation), sequencing technology (Illumina MiSeq), and especially the analysis of the obtained sequencing reads are crucial factors in the success of the aforementioned studies. The NetoVIR protocol, developed primary for the study of the human microbiota but also considered useful for other tissue samples, offers a simple modular approach for the preparation of samples prior to Illumina sequencing, combining homogenization, centrifugation, and filtration [40]. Although a strong increase in the ratio of viral reads, up to 96.7% of the total number of reads, is reported, the protocol has only been optimized and proven useful for fecal samples and not yet for solid-organ material, which contains a higher rRNA background. Wylezich et al. developed a metagenomics processing workflow based on cryoPREP^®^ disruption, suitable for the detection of pathogens in animal, human, and food samples [41]. When performed prior to deep genome sequencing using the RIEMS platform, in some cases with over 2 million total reads per sample [42], various known and novel pathogens were identified in a wide variety of sample matrices, including solid tissues. However, this result was obtained with low numbers and very small proportions of viral reads [41,43,44]. In none of the published workflows was an enrichment step included that would drastically lower the number of reads that had to be processed through metagenomics and, therefore, our study shows the first approach for increasing sensitivity without the need of more sequencing space.

The major contaminant of homogenized tissues and major contributor to background sequences, rRNA, could not be completely removed from homogenates. The structural stability of ribosomes, which contain the competing rRNA for NGS virus detection, along with their similar size to viruses (>700 kDa), impedes their physical separation using size exclusion chromatography methods such as Capto™ Core 700 [7,13]. Nonetheless, the combination of EDTA treatment, sonication, organic extraction, and chromatography removed significant amounts of competing nucleic acids, which also take up sequencing space. Other studies have advocated the use of commercially available kits for the removal of rRNA. Rosseel et al. showed how the use of the Ribo-Zero Magnetic Gold Epidemiology kit (Epicentre Technologies, Thane, India) for the rRNA depletion in chicken lung tissue resulted in the identification of increased numbers of Newcastle disease virus reads [13]. Although successful, the price per sample was high (70 € in 2015 when it was published), undoubtedly a significant drawback when larger numbers of tissue samples need to be tested during an outbreak. Our suggested semi-high-throughput enrichment method has an estimated cost per sample of 10 €.

Our study is the first to show that enrichment of homogenized tissue samples results in reduction in total sequence reads combined with an increased sensitivity towards viral reads in VIDISCA. It is important to note that obtaining a higher absolute number of viral reads to perform full genome sequencing is not the goal when using VIDISCA. The primary aim is to have an increased chance of finding a segment of a (novel) viral pathogen. When identified, other molecular biology techniques and sequencing methods are at the disposal of the researcher for full genome detection and virus isolation. Existing NGS methods for virus detection use very large sequencing space, allowing detection of as many reads as possible: the deeper you search, the more chance you have of finding something. Nevertheless, when using organ samples, even deep sequencing usually hits just a few reads due to the excessive amounts of contaminating nucleotides present in those samples [41]. The described workflow shows that we can free up sequencing space by removing this contaminating material (primarily non rRNA), allowing for an easier detection and analysis of viral reads in tissue material and subsequent full identification using other methods.

In conclusion, when processing of tissue homogenates with both EDTA 25 mM and Vertrel™ XF prior to sonication is combined with subsequent Capto™ Core 700 enrichment, there is a beneficial decrease of background reads compared to viral reads, resulting in increased sequencing space and augmented sensitivity of the VIDISCA-NGS method. The workflow enables new virome studies, especially in the veterinary field, by including organ homogenates in the regular high throughput diagnostic analysis of animal samples (i.e., test a certain proportion of samples among the total of samples obtained in animal experiment or field studies). Subsequently the results could be further corroborated by PCR analysis on the samples, i.e., a PCR designed specifically on the novel sequence identified, allowing for rapid testing of multiple samples obtained in field studies. One can later decide, with the new information, what methods would be preferred to further identify and characterize the novel viral pathogen. This combined assessment of body fluids and tissue materials from necropsied animals is especially relevant in situations when regular diagnostics fail to explain disease or when new disease presentations with multiple involved pathogens are recognized. The easy processing workflow expands applicability of NGS platforms for the discovery, characterization, and study of infectious pathogens. Furthermore, it enables simultaneous sample processing in a short time frame of only a few hours, and the required instruments are accessible in the majority of laboratories. Importantly, fewer reads need to be analyzed and the computational power needed is accordingly less, which reduces the economic costs and increases the number of samples that can be tested in one sequencing run.

## Figures and Tables

**Table 1:** Experimental processing workflows performed prior to VIDISCA.

Pre-Treatment	Workflow 1	Workflow 2	Workflow 3	Workflow 4
Sonication	yes	yes	yes	yes
EDTA 25 Mm ^1^	no	yes	no	yes
Vertrel™ XF ^2^	no	no	yes	yes

^1^ Final concentration of EDTA in the sample was 25 mM. ^2^ Vertrel™ XF equal to sample volume.

**Table 2:** APPV and rRNA qPCR results after sample processing with the different workflows and number of chromatography rounds.

Chromatography Rounds	APPV Copies/µL		APPV Cq Values		rRNA Cq Values	
Replicates	1	2	1	2	1	2
Initial material	5.56 × 10^4^	1.61 × 10^4^	20.61	22.5	14.31	12.28
**Workflow 1 (sonication only)**						
0	1.60 × 10^4^	1.33 × 10^4^	22.35	22.78	13.82	14.74
1	3.64 × 10^4^	4.87 × 10^3^	21.20	24.29	13.95	12.76
2	6.14 × 10^4^	2.81 × 10^3^	20.46	25.12	14.76	12.00
3	4.72 × 10^4^	2.40 × 10^3^	20.82	25.36	12.50	12.62
**Workflow 2 (EDTA 25 mM followed by sonication)**						
0	6.96 × 10^4^	8.44 × 10^3^	20.27	23.49	15.27	16.37
1	6.55 × 10^4^	1.62 × 10^3^	20.35	25.96	16.24	16.88
2	2.09 × 10^4^	1.37 × 10^3^	21.95	26.21	17.78	17.59
3	2.32 × 10^4^	1.39 × 10^2^	21.79	29.63	18.64	16.78
**Workflow 3 (Vertrel™ XF followed by sonication)**						
0	4.88 × 10^3^	9.35 × 10^2^	24.01	26.75	16.94	17.99
1	2.63 × 10^4^	3.36 × 10^2^	21.65	28.28	16.29	13.17
2	2.96 × 10^4^	9.41 × 10^2^	21.48	26.75	14.75	10.94
3	1.59 × 10^4^	4.91 × 10^2^	22.35	27.72	15.96	12.63
**Workflow 4 (EDTA 25 mM and Vertrel™ XF followed by sonication)**						
0	1.88 × 10^4^	8.32 × 10^2^	22.10	26.94	17.86	20.13
1	1.29 × 10^4^	1.56 × 10^2^	22.63	29.44	17.99	21.23
2	6.00 × 10^3^	4.17 × 10^1^	23.69	31.42	19.88	21.92
3	3.59 × 10^3^	1.41 × 10^2^	24.40	29.61	19.94	22.63

**Table 3:** Sequence reads of 18s RNA, 28s RNA, and APPV in VIDISCA-NGS libraries after sample processing and enrichment with the different workflows.

Chromatography Rounds	Total Number of Reads		rRNA Reads		% Reads rRNA		APPV Reads		% Reads APPV	
	Replicate (R) 1	R2	R1	R2	R1	R2	R1	R2	R1	R2
Initial material	84,084	63,854	25,356	21,257	30.16	33.29	5	8	0.006	0.013
**Workflow 1 (sonication only)**										
0	84,778	53,634	21,700	10,392	25.60	19.38	8	11	0.009	0.021
1	68,775	25,915	23,385	24,967	34.00	96.34	5	5	0.007	0.019
2	40,924	25,840	34,588	25,626	84.52	99.17	5	1	0.012	0.004
3	75,399	42,923	73,252	42,254	97.16	98.44	3	3	0.004	0.007
**Workflow 2 (EDTA 25 mM followed by sonication)**										
0	46,792	54,151	31,863	21,555	68.10	39.81	10	66	0.021	0.122
1	33,707	43,218	29,486	15,124	87.48	34.99	3	17	0.009	0.039
2	34,984	52,576	27,840	18,457	79.58	35.85	4	14	0.011	0.027
3	31,350	34,361	25,633	28,467	81.76	82.85	7	5	0.022	0.015
**Workflow 3 (Vertrel™ XF followed by sonication)**										
0	63,635	62,708	30,146	4390	47.37	7.00	11	2	0.017	0.003
1	31,928	45,075	30,578	44,168	95.77	97.99	10	1	0.031	0.002
2	29,822	29,509	29,222	29,116	97.99	98.67	3	0	0.010	0
3	45,833	25,810	44,714	25,571	97.56	99.07	0	2	0	0.008
**Workflow 4 (EDTA 25 mM and Vertrel™ XF followed by sonication)**										
0	31,579	16,361	29,564	2631	93.62	16.08	8	12	0.025	0.073
1	22,249	4508	19,821	769	89.08	17.06	6	1	0.027	0.022
2	6329	4177	4423	1043	69.89	24.97	4	5	0.063	0.120
3	1944	954	1204	265	61.93	27.78	1	1	0.051	0.105

## Data Availability

Not applicable.

## Supplementary Materials

The following are available online at https://www.mdpi.com/article/10.3390/diagnostics11050791/s1, Table S1. Spleen and brain tissues from the same necropsied animals were processed using workflow 4 followed by two Capto™ Core 700 purification rounds; Figure S1. The effect of Vertrel™ XF organic extraction, EDTA treatment and Vertrel™ XF organic extraction combined with EDTA treatment on the activity of RT-qPCR enzymes was tested on HCoV-NL63 samples; Figure S2. The effect of Vertrel™ XF organic extraction, EDTA treatment, and Vertrel™ XF organic extraction combined with EDTA treatment on the activity of TURBO™ DNase enzyme was tested on a Lambda DNA HindIII digested marker.
