# Supplementary material for: A Versatile Processing Workflow to Enable Pathogen Detection in Clinical Samples from Organs Using VIDISCA
Source: Diagnostics (Basel). 2021 Apr 27;11(5):791. doi: 10.3390/diagnostics11050791 (PMC8145099; doi:10.3390/diagnostics11050791)
Supplement: Supplementary file 1 [file diagnostics-11-00791-s001.zip › diagnostics-1126760-supplementary.pdf]

Supplementary Materials

A Versatile Processing Workflow to Enable Pathogen Detection in Clinical Samples from Organs Using VIDISCA

Sample		Total Reads	rRNA Ct-Value	Virus Detected	Read Number
Organ	Species				
**Spleen**	Swine	2858	20.16	APPV	3
**Brain**	Swine	19634	18.72	APPV	1

Table S1. Spleen and brain tissues from the same necropsied animals were processed using workflow 4 followed by two Capto™ Core 700 purification rounds. The protocol allowed detection of APPV via VIDISCA-NGS.

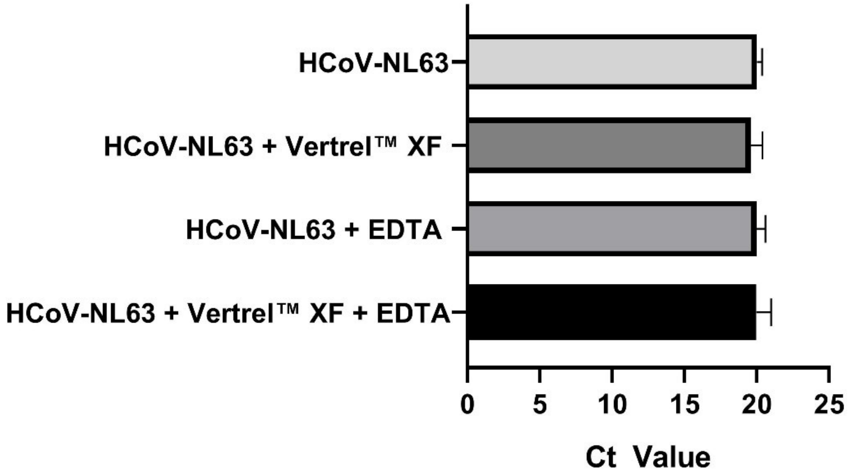

Figure S1. The effect of Vertrel™ XF organic extraction, EDTA treatment and Vertrel™ XF organic extraction combined with EDTA treatment on the activity of RT-qPCR enzymes was tested on HCoV-NL63 samples. The graph shows the mean Ct value and standard deviation of the samples tested. No statistically significant difference was found between the treatment conditions and the control (One-way ANOVA; *p*-value = 0.8886).

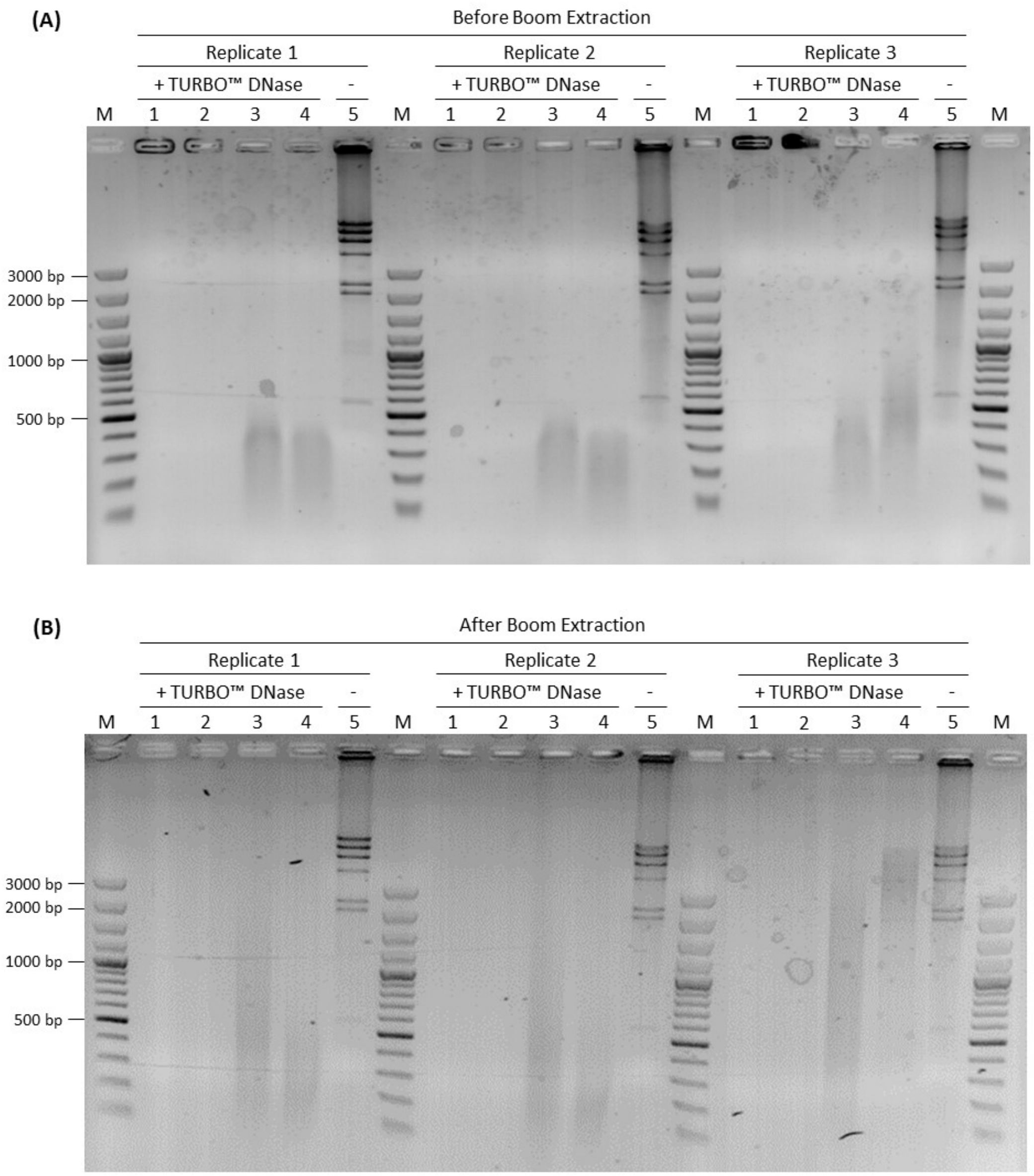

Figure S2. The effect of Vertrel™ XF organic extraction, EDTA treatment, and Vertrel™ XF organic extraction combined with EDTA treatment on the activity of TURBO™ DNase enzyme was tested on a Lambda DNA HindIII digested marker. Experiments were performed in triplicate. (**A**) Samples before Boom extraction. (**B**) Samples after Boom extraction. M: DNA ladder; 1: untreated Lambda DNA HindIII digested marker; 2: Lambda DNA HindIII digested marker + Vertrel™ XF; 3: Lambda DNA HindIII digested marker + EDTA; 4: Lambda DNA HindIII digested marker + Vertrel™ XF + EDTA; 5: Lambda DNA HindIII digested marker without TURBO™ DNase treatment.
